# Cerebral Air Embolism After Endoscopy: A Case Report

**DOI:** 10.5811/cpcem.1371

**Published:** 2024-01-09

**Authors:** James Whall, Eli Besser, Jaymes Lonzanida, Ryan Roten

**Affiliations:** * Desert Regional Medical Center, Department of Emergency Medicine, Palm Springs, California

**Keywords:** *case report*, *cerebral air embolism*, *endoscopy*

## Abstract

**Introduction:**

Cerebral air embolisms are a rare but potentially devastating event where air enters the vascular system. Although commonly associated with intravascular catheters, they can arise from a variety of mechanisms including endoscopic procedures.

**Case Report:**

We report the case of a 90-year-old woman who presented with focal neurologic deficits due to an air embolism after undergoing an esophagogastroduodenoscopy.

**Conclusion:**

Cerebral air embolisms should be considered in patients who present to the emergency department with acute neurologic changes, especially after an endoscopic procedure.

Population Health Research Capsule What do we already know about this clinical entity?

*Cerebral air embolisms are a rare but potentially devastating condition caused by air entering the cerebral vasculature; they often present with stroke-like symptoms.*

What makes this presentation of disease reportable?

*Cerebral air embolism after esophagogastroduodenoscopy is extremely rare with only 13 previously reported cases.*

What is the major learning point?

*Air embolism should be suspected in any patient with acute neurologic changes after an endoscopic procedure. Treatment is hyperbaric oxygen therapy and repositioning.*

How might this improve emergency medicine practice?

*Awareness of this rare but serious disease process will lead to more rapid identification and treatment.*

## INTRODUCTION

Air embolisms are characterized as unwanted air in the vascular system with potentially devastating morbidity and mortality.^1^ More commonly associated with intravascular catheters, these events have been rarely reported in a variety of endoscopic procedures.^2^ Although there are potentially devastating consequences of cerebral air embolisms (CAE), rapid diagnosis and treatment improves chances of recovery.^3^ Therefore, this rare complication should be considered in patients presenting for focal neurological deficits after endoscopic procedure.

## CASE REPORT

A 90-year-old woman presented to the emergency department (ED) from an outpatient surgical center after undergoing an esophagogastroduodenoscopy (EGD) with right gaze deficit, left upper extremity flaccid paralysis, and aphasia. The patient received fentanyl and midazolam for sedation during the EGD, which were reversed with naloxone and flumazenil prior to arrival to the ED.

**Image 1.**
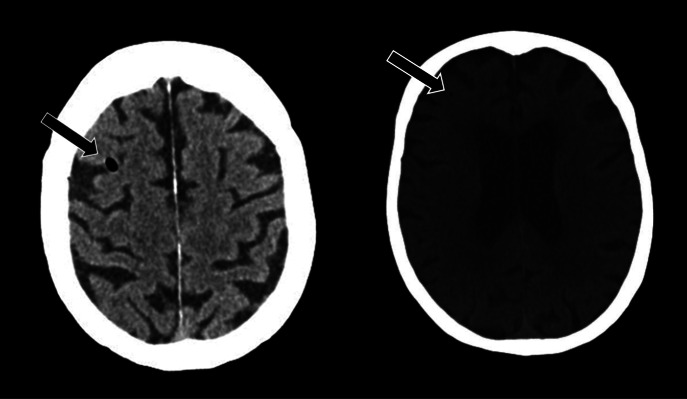
Computed tomography head without contrast demonstrating air embolisms (arrow).

The patient’s presenting vital signs were a temperature of 36.8° Celsius, heart rate of 75 beats per minute, respiratory rate of 16 breaths per minute, blood pressure of 130/75 millimeters of mercury, and pulse oximetry at 100% on room air. The physical exam was significant for forced gaze to the right, left lower facial weakness, left upper and lower extremity flaccid paralysis. The patient was able to intermittently follow commands but could not speak. She was rapidly taken for imaging. A computed tomography (CT) head without contrast found several sub-centimeter air embolisms in the right frontal parietal region (Image 1).

**Image 2.**
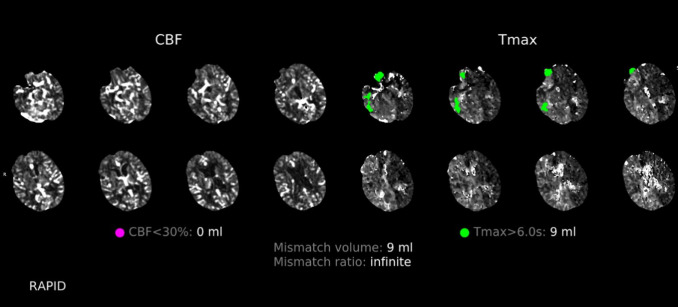
Computed tomography head with contrast demonstrating right frontal, parietal, and temporal regions with perfusion deficits (green). These deficits are defined by a time-to-maximum delay of over six seconds for contrast to move to the tissue. Without intervention, these regions are a reliable estimate of the final infarction area. There are no regions with cerebral blood flow deficits (purple), which would indicate completed ischemia.^4^ *CBF*, cerebral blood flow; *Tmax*, time to maximum.

There was no evidence of intracranial hemorrhage or mass effect. A CT angiography of the head and neck did not reveal large vessel occlusions. A CT head with contrast found scattered right frontal, parietal, and temporal perfusion abnormalities compatible with ischemia (Image 2). Upon her return from imaging, the patient had seizure activity with rhythmic movement of the right upper extremity. The patient was treated with multiple doses of benzodiazepines and levetiracetam. She ultimately required intubation and was placed on a continuous infusion of midazolam.

Given the CT findings of CAE, the patient was placed in the left lateral Trendelenburg position, the fraction of inspired oxygen (FiO_2_) was maximized to 100%, and dexamethasone was given upon recommendation from the neurologist. A transthoracic echocardiogram found a normal ejection fraction, no patent foramen ovale, and no other significant findings. An electroencephalogram found no evidence of non-convulsive status epilepticus. The patient was transferred to a higher level of care for hyperbaric oxygen (HBO) therapy. Per the patient’s family, the patient did not have improvement at the tertiary hospital and was placed on comfort care.

## DISCUSSION

Cerebral air embolisms have been reported in a variety of endoscopic procedures; however, they remain uncommon. A recent review of inpatient procedures found a rate of 0.57/100,000 endoscopic procedures and specifically 0.44/100,000 for EGD.^5^ A review of recent literature found a case of CAE presenting as a tonic-clonic seizure in a 52-year-old man during an EGD and a 71-year-old man presenting with hemiparesis and dysarthria two hours after undergoing EGD.^6^ ^,^ ^7^ In total there appear to be only 13 reported cases of CAE after EGD, reinforcing its rarity.^8^

There are a variety of proposed mechanisms by which gas can enter vasculature during endoscopy including through the portal vein, exposed gastrointestinal vessels, or through adjacent veins of inflamed mucosa.^2^ Theories on how air reaches the arterial system, and specifically the central nervous system, include paradoxical embolization through heart shunts such as a patent foramen ovale, retrograde flow through the superior vena cava, and through pulmonary veins if not filtered by the pulmonary system.^8^ The air then causes ischemia and injury by directly occluding vessels or by initiating an inflammatory cascade leading to thrombus formation.^9^

Although exceedingly rare, air embolism should be suspected in any patient with acute neurologic changes after an endoscopic procedure. A CT head may show evidence of cerebral gas embolism, as in this case. Gas may also be reabsorbed quickly and not be evident on imaging. Treatments include the following: 100% FiO_2_ to reduce bubble volume and increase diffusion gradient; placement in the left lateral Trendelenburg position; and HBO.^3^ Hyperbaric oxygen therapy is the most critical intervention; if started within five hours, HBO can double the chances of full recovery.^10^

## CONCLUSION

Air embolisms are a rare but potentially devastating event. Air embolisms should be considered in patients who present to the ED with acute neurologic changes, especially after an endoscopic procedure. Rapid identification and treatment can lead to improved outcomes for the patient, highlighting the importance of increased awareness of this condition.

## References

[1] Lanke G Adler DG. Gas embolism during endoscopic retrograde cholangiopancreatography: diagnosis and management. Ann Gastroenterol. 2019;32(2):156–67. 30837788 10.20524/aog.2018.0339 PMC6394273

[2] Donepudi S Chavalitdhamrong D Pu L et al. Air embolism complicating gastrointestinal endoscopy: a systematic review. World J Gastrointest Endosc. 2013;5(8):359–65. 23951390 10.4253/wjge.v5.i8.359 PMC3742700

[3] Cooper J. Treatment of endoscopy associated cerebral gas embolism. Am J Gastroenterol. 2018;113(12):1742–4. 29915395 10.1038/s41395-018-0139-z PMC6768578

[4] Butcher K Parsons M Allport L et al. Rapid assessment of perfusion–diffusion mismatch. Stroke. 2008;39(1):75–81. 18063829 10.1161/STROKEAHA.107.490524

[5] Olaiya B Adler DG. Air embolism secondary to endoscopy in hospitalized patients: results from the National Inpatient Sample (1998–2013). Ann Gastroenterol. 2019;32(5):476–81. 31474794 10.20524/aog.2019.0401 PMC6686097

[6] Popa D Grover I Hayden S et al. Iatrogenic arterial gas embolism from esophagogastroduodenoscopy. J Emerg Med. 2019;57(5):683–8. 31672399 10.1016/j.jemermed.2019.08.053

[7] Pandurangadu AV Paul JAP Barawi M et al. A case report of cerebral air embolism after esophagogastroduodenoscopy: diagnosis and management in the emergency department. J Emerg Med. 2012;43(6):976–9. 21236613 10.1016/j.jemermed.2010.11.031

[8] Farouji I Chan KH Abed H et al. Cerebral air embolism after gastrointestinal procedure: a case report and literature review. J Med Cases. 2021;12(3):119–25. 34434442 10.14740/jmc3639 PMC8383579

[9] Chuang DY Sundararajan S Sundararajan VA et al. Accidental air embolism. Stroke. 2019;50(7):e183–6. 31164070 10.1161/STROKEAHA.119.025340

[10] Murphy BP Harford FJ Cramer FS. Cerebral air embolism resulting from invasive medical procedures. Treatment with hyperbaric oxygen. Ann Surg. 1985;201(2):242–5. 3918516 10.1097/00000658-198502000-00019 PMC1250649

